# Social integration and its value within the multiple setting in stroke care

**Published:** 2012-09-04

**Authors:** Nina Szczygiel, Silvina Santana

**Affiliations:** Assistant Lecturer and Researcher, Research Unit on Governance, Competitiveness and Public Policies, Department of Economics, Management and Industrial Engineering, University of Aveiro, Aveiro, Portugal; Associate Professor with Aggregation, Institute of Electronics Engineering and Telematics of Aveiro, Department of Economics, Management and Industrial Engineering, University of Aveiro, Aveiro, Portugal

**Keywords:** social integration, networks, quality of life, stroke

## Abstract

**Purpose:**

To report the scope and illustrate the relevance of social integration among stroke patients.

**Theory:**

Modern understanding of health has evolved over time and so nowadays aspects of health have moved beyond of what the health sector can handle on its own. Social integration is increasingly deemed to be associated with health outcomes and social networks and interactions with relatives have been considered important predictors of quality of life.

In 2006 the National Network of Continuous Integrated Care (RNCII) was established in Portugal, aiming, among others, to bridge the gap in interactions between health and social services. Nevertheless, and despite some acknowledged successes, the system maintains fragmented, especially in what the home care phase (after the acute and the eventual institutional convalescence phase) concerns. Social isolation, especially in older adults, the most subjected into stroke incidents at the same time, is currently considered a relevant and a very realistic threat to a person’s well-being. While for the elderly performing daily tasks and activities and driving within the health and social care system may already be difficult, social integration seems to be even more challenging, through emotional, psychological and motor limitations.

**Methods:**

The study is based on Portuguese patients admitted to a stroke unit, randomized through an RCT into the intervention or the control group. Patients were followed during 6 months after discharge. The Lubben Social Network Scale-18 (LSNS-18) was applied, assessing perceived social support received from family, neighbors and friends. The intent behind this was to bring together and review strength, deepness, perceived availability and reliability of these interactions.

**Results and conclusions:**

The conducted analysis established the dimension of social integration of stroke patients. Patients reported to have reasonably strong relationships with kin and non-kin, with a major role of family members. However, in line with what has been generally spoken in the public debate in recent years and with the perception of professionals engaged in the study, the traditional model of family and closeness of relationships in Portugal are fading away. We identified a number of patients who could not count on any family member, but yes, on non-kin, as well as patients who were left in a total solitude.

**Discussion:**

Literature suggests that social networks are positively associated with functioning in several groups of patients. Social integration should then be considered by policy-makers and by health and social care programmes’ and initiatives’ designers. We conclude stressing the contribution of the society awareness for this issue.

